# ﻿Hidden treasures of herbaria - even small collections contain a wealth of diversity: the powdery mildews of the North Carolina State Larry F. Grand Mycological Herbarium

**DOI:** 10.3897/imafungus.16.156231

**Published:** 2025-06-10

**Authors:** Scott LaGreca, Uma Crouch, Andrew Paul, Jacklyn Thomas, Jake Thompson, Christian Shaw, Marc A. Cubeta, Uwe Braun, Michael Bradshaw

**Affiliations:** 1 Department of Entomology and Plant Pathology, Center for Integrated Fungal Research, North Carolina State University, Raleigh, NC, 27606, USA North Carolina State University Raleigh United States of America; 2 Sarah P. Duke Gardens, 420 Anderson Street, Duke University, Durham, NC, 27708-0341, USA Duke University Durham United States of America; 3 Department for Geobotany and Botanical Garden, Institute of Biology, Martin Luther University, Herbarium, Halle (Saale), 06108, Germany Martin Luther University Halle Germany; 4 Farlow Herbarium, Harvard University, 22 Divinity Avenue, Cambridge, MA 02138, USA Harvard University Cambridge United States of America

**Keywords:** Collection Based Research, *Erysiphaceae*, Fungarium, Museums, NCSLG

## Abstract

The occurrence of cryptic species is well documented in fungi but the extent of their diversity is not fully understood. This study assessed the fungal diversity within a part of the Larry F. Grand Mycological Herbarium (NCSLG), a small, well-maintained collection at North Carolina State University, with a focus on the powdery mildew fungi (*Erysiphaceae*). *Erysiphaceae* were selected due to their economic impact as plant pathogens and availability of extensive DNA sequence data for multiple barcode loci. Our research objectives included determining the number of phylogenetic species compared with those identified morphologically, and to identify undescribed species. We generated sequence data for 220 of the 299 powdery mildew specimens (73% success rate) in the herbarium, which represented 60 species in 10 genera, collected from 134 host plant species. Our analyses revealed that ~83% (183/220) of the sequenced specimens had identifications that were incorrect and/or outdated based on current genus/species concepts. Additionally, four new species are described: *Erysipheamphicarpaeicola*, *E.ulmi-alatae*, *E.quercus-virginianae*, and *Takamatsuellagrandii*. A specimen deposited at NCSLG is designated as an epitype for *Phyllactinialiriodendri*, and a species of *Phyllactinia* identified on *Carpinuscaroliniana*, as well as multiple species infecting *Quercus* spp., likely represent additional undescribed species that require more data. This research highlights the critical role of herbarium collections in uncovering fungal biodiversity, and underscores the importance of preserving these valuable resources, particularly with the growing trend to discard herbaria due to financial and space constraints.

## ﻿Introduction

Herbaria provide critical infrastructure for addressing key scientific research questions (Lavoie 2013; Dentinger et al. 2015; Verkley et al. 2015; James et al. 2018). Traditionally, they have served as a reference for biodiversity research, a field of biological inquiry that relies on accurate species identification. Recent studies have demonstrated that herbarium specimens represent a rich yet underutilized resource to better understand fungal evolution (Staats et al. 2013; Dentinger et al. 2016; Tedersoo et al. 2016). More modern, non-traditional uses of herbaria include tracking species of fungi and plants through time and space to provide evidence of climate and environmental change as well as to understand host-pathogen interactions and disease spread (Lendemer et al. 2017; Willis et al. 2017; Meineke et al. 2018; Kido et al. 2019; Bradshaw et al. 2025c). Additionally, the increasing digitization of herbarium specimens has significantly enhanced their role in global biodiversity research, particularly through platforms like GBIF, which now serve as critical infrastructures for data accessibility (Knapp 2023; Park et al. 2023). Even though strides have been made to bring herbarium research into the 21^st^ century (Staats et al. 2013; Detinger et al. 2015; Yoshida et al. 2015; Buerki et al. 2016; Tedersoo et al. 2016; Forin 2018; Miller et al. 2022), their importance in research is broadly underutilized and undervalued (Andrew et al. 2018). Originally established to serve applied purposes—such as documenting medicinal plants —herbaria continue to hold untapped potential for addressing contemporary societal needs, including applications in public health, agriculture, education, and other translational research areas.

The influence of herbaria goes beyond scientific endeavors with herbarium specimens inspiring artwork as well as providing a unique resource for historians developing biographies of plant collectors (Flannery 2023). Another innovative use for herbaria is mining preserved specimens for DNA to study population genetics and reconstruct evolutionary relationships (Bradshaw and Tobin 2020; Bradshaw et al. 2021, 2023a, 2025c; Ristaino 2020; Folk et al. 2021). Recent technological advances have improved extraction, amplification, and sequencing of DNA from historic fungal material (Kistenich et al. 2019; Bradshaw et al. 2023a). Because of these advances, fungal herbaria (fungaria) represent an underutilized source of historical DNA that is now being used to explore many interesting research questions, from tracking the evolution and spread of plant disease-causing fungi and fungal-like organisms (Ristaino and Schmidt 2014; Bradshaw et al. 2021, 2025c), to revealing patterns of host adaptation and specialization of plant pathogens (Bradshaw et al. 2024, 2025c), to discovering undescribed fungal species (Bradshaw et al. 2025a, 2025b).

The Larry F. Grand Mycological Herbarium (official acronym: NCSLG; NYBG Steere Herbarium 2024) is a small (c. 14,000 specimens) fungal herbarium located in Gardner Hall on the North Campus of North Carolina State University in Raleigh. The herbarium was established in 1970 by Professor Larry F. Grand (1940–2013), a mycologist who specialized in the study of wood decay, plant pathogenic, and ectomycorrhizal fungi. Professor Grand’s research interests are strongly reflected in the fungarium. At the time of his retirement, wood decay fungi comprised over half the collections, followed by plant pathogenic and ectomycorrhizal mushroom forming fungi. Roughly, the collection consisted of 75% *Basidiomycota* and 25% *Ascomycota*, with a few specimens of zygomycetous fungi, *Oomycetes*, and slime molds. Geographically, most specimens were collected from the Southeastern United States. Unique North Carolina habitats of conservation concern (Schafale 2024) represented in the NCSLG collection include bald regions of the Southern Appalachian Mountains and Nags Head Woods, a remnant Coastal Plain longleaf pine ecosystem. NCSLG also houses a substantial library of more than 1000 mycology books, scientific journals, illustrations, and reprints. Although Dr. Grand’s mycological expertise was wood decay fungi, he had a major interest in plant pathogenic fungi such as the powdery mildew and rust fungi. During Grand’s tenure as Director of the herbarium, he accumulated ~300 powdery mildew specimens.

Cryptic species represent morphologically indistinguishable, monophyletic species and are well-documented for fungi (Groenewald et al. 2006; Schubert et al. 2007; Bensch et al. 2010; Crous et al. 2012; Videira et al. 2016; LaGreca et al. 2020; U’Ren et al. 2024). Hawksworth and Lücking (2017) estimated that the Kingdom Fungi comprises between 2.2 and 3.8 million species, with a substantial number “hidden” as cryptic species. As a first step in exploring the hidden diversity present within a small, state university fungal herbarium, representative loci (Bradshaw et al. 2022) were sequenced for the powdery mildew (*Erysiphaceae*) specimens in NCSLG. The *Erysiphaceae* was chosen as our study group of fungi because of their economic importance as plant pathogens and the availability of DNA sequence data for multiple barcode loci (Bradshaw et al. 2022). Our primary research objective was to determine and compare the number of distinct phylogenetic species based on DNA analyses to the number of species names based on specimen collection labels. Given the large diversity of powdery mildew hosts in the herbarium, we hypothesized that our analyses of the powdery mildews in the herbarium would reveal multiple undescribed species.

## ﻿Materials and methods

### ﻿Morphological examination

Leaves and stems of each powdery mildew herbarium specimen were initially examined for mycelium, conidia and conidiophores, and appendages, asci, ascospores, and peridial cells of chasmothecia with a Nikon SMZ1270 dissecting microscope. To examine asexual structures, a 2 × 4 cm piece of 3M clear, adhesive tape was applied to the visible colonies of powdery mildew fungi on a diseased leaf, followed by placing the tape on a drop of distilled water or 3% KOH on a glass slide. The following measurements were taken: width of hyphae, length/width of conidiophores, and length/width of conidia (n = 20 for each structure, unless otherwise noted). To examine chasmothecia, a sterile dissecting needle was used to transfer c. 10 chasmothecia from diseased leaves (when possible) to a glass slide with a droplet of 10% KOH for a period of 5 minutes to allow for rehydration. The rehydrated chasmothecia were then transferred to a glass slide with a droplet of water followed by a glass cover slip. The following measurements were made: chasmothecium diameter, length/width of peridial cells, length/width of chasmothecial appendages, length/width of asci, length and width of asci, and length/width of ascospores (n = 20 for each structure, unless otherwise noted). Slides were examined with a Nikon Eclipse E600 compound microscope at 10×, 40 × and 100 × (= oil immersion) magnifications. Images were captured with a Nikon DSRi2 digital camera attached to the microscopes. All measurements were made using dedicated Nikon NIS-Elements 5.42.04 software. For the conidia of *E.ulmi-alatae* pen and ink hand illustrations were accomplished by the first author.

### ﻿DNA sequencing

DNA extractions were performed using the Chelex method (Walsh et al. 1991; Hirata and Takamatsu 1996). The polymerase chain reaction (PCR) was used to amplify the ITS and LSU rDNA regions using the primer pairs PM10/PM28R (Bradshaw and Tobin 2020) for all specimens. If PCR was unsuccessful, a nested approach was applied using primers AITS (Bradshaw and Tobin 2020)/TW14 (Mori et al. 2000) followed by PM10/PM28R or AITS/PM11 (Bradshaw and Tobin 2020) followed by PM10/PM2 (Cunnington et al. 2003). For the undescribed species and for taxa that needed additional data for accurate identification, multiple loci were sequenced. For the calmodulin (*CAM*), Glyceraldehyde 3-Phosphate Dehydrogenase (*GAPDH*), Glutamine synthetase (*GS*), and RNA Polymerase II Subunit B2 (*RPB2*) region the primer pairs PMCAM1/PMCAM4R, PMGAPDH1/PMGAPDH3R, GSPM2/GSPM3R, and PMRpb2_4/PMRpb2_6R were used (Bradshaw et al. 2022). If these amplifications were unsuccessful for the *GS* and *RPB2* regions, the following primers from Bradshaw et al. (2023b) were used: EGS1/EGS2R and ERPB2_3/ERPB2_7R. For the ß-tubulin (*TUB*) region the primers BTF5b/BTR7a (Ellingham et al. 2019) were used followed by ETUB2 and ETUB2R (Bradshaw et al. 2023b). In reactions where *GAPDH* sequences were contaminated with DNA of *Ampelomyces* mycoparasites, EGAPDH1/EGAPDH2 primers (Bradshaw et al. 2025a) were used. To amplify the rDNA intergenic spacer (IGS) region, the primer pair IGS-12a/NS1R was used (Carbone and Kohn 1999).

### ﻿Phylogenetic analysis

Phylogenetic trees were generated from analysis of concatenated ITS+28S+*CAM*+*GAPDH*+*GS*+IGS+*RPB2*+*TUB* sequences. Sequences were aligned and edited using Geneious Prime 2025.0.3. Taxa were chosen based on the analyses by Bradshaw et al. (2023b, 2025d). A GTR+G+I evolutionary model was used for phylogenetic analyses as it is the most inclusive model of evolution and includes all other evolutionary models (Abadi et al. 2019). The phylogeny was inferred using Bayesian analysis of the combined loci using a Yule tree prior (Gernhard 2008) and a strict molecular clock, in the program BEAST version 1.10.4 (Suchard et al. 2018). A single MCMC chain of 10^7^ steps was run, with a burn-in of 25%. Posterior probabilities were calculated from the remaining 9000 sampled trees. A maximum clade credibility tree was produced using TreeAnnotator version 1.10.4 (part of the BEAST package). Stationarity was confirmed by running the analysis multiple times, which revealed convergence between runs. The resulting tree was visualized using FigTree ver. 1.3.1 (Rambaut, 2009). A maximum likelihood analysis was accomplished using raxmlGUI (Silvestro and Michalak 2012) under the default settings with a GTR+G+I evolutionary model. Bootstrap analyses were conducted using 1000 replications (Felsenstein 1985).

## ﻿Results

**Figure 1.**
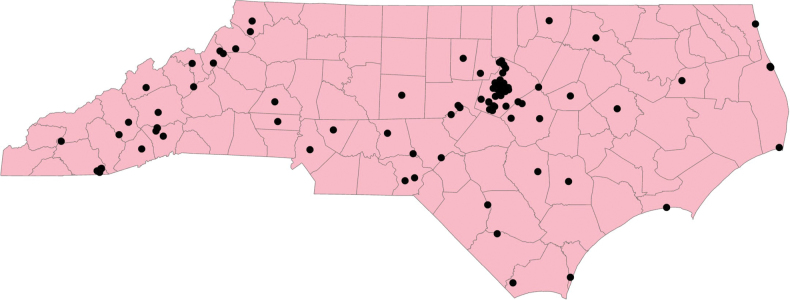
Map of North Carolina showing collection localities of *Erysiphaceae* specimens in the NCSLG herbarium. The cluster of collections in the middle of the state, representing ~66% of our specimens, is from the NCSU campus and surrounding Raleigh area.

**Figure 2.**
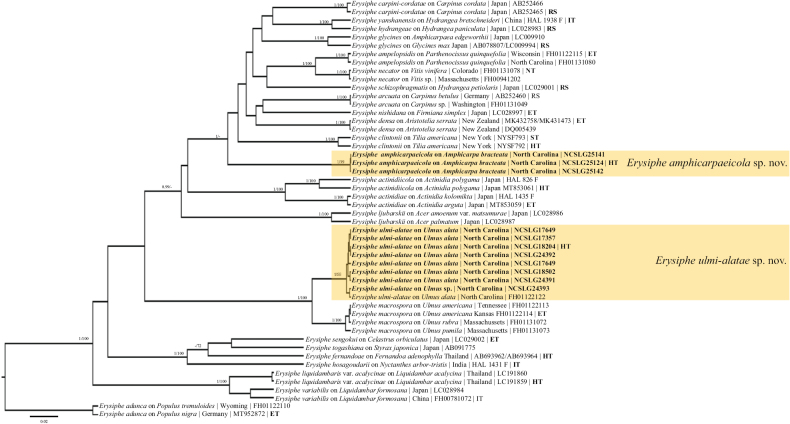
Bayesian maximum clade credibility tree of concatenated ITS+28S+*CAM*+*GAPDH*+*GS*+IGS regions of select taxa in the *Uncinula* lineage. The phylogenetic tree revealed two undescribed species. Fungal species are denoted and followed by the host, collection locality, and voucher number. Type status (HT: Holotype, IT: Isotype, ET: Epitype, NT: Neotype, ST: Syntype) of the specimens concerned is denoted as well as reference sequences for phylogenetic purposes (RS). Posterior probabilities ≥ 90 are displayed followed by bootstrap values greater than 70% for the maximum likelihood (ML) analyses. Taxa in bold were sequenced for the current study.

**Figure 3.**
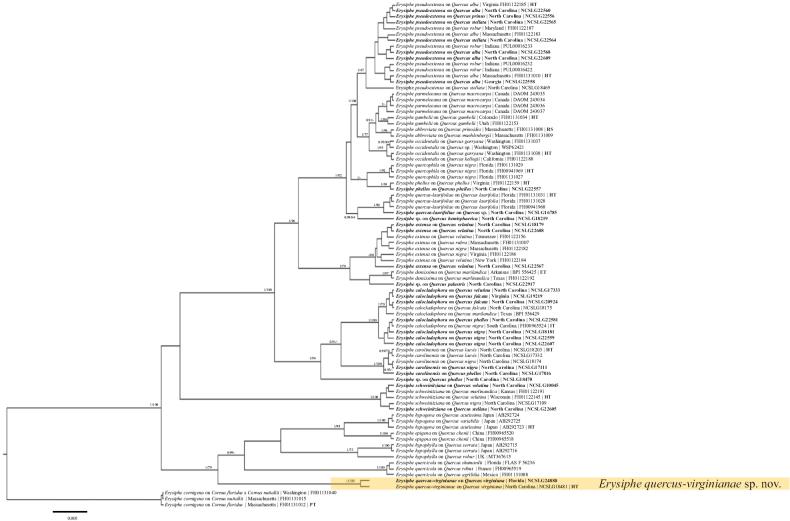
Bayesian maximum clade credibility tree of the concatenated ITS+28S+*CAM*+*GAPDH*+*GS*+IGS+*RPB2*+*TUB* regions of taxa in the North American *Quercus* lineage. The phylogenetic tree revealed one undescribed species as well as multiple, additional undescribed species, labeled as ‘*Erysiphe* sp.’ Fungal species are denoted followed by the host, collection locality, and voucher number. Type status (HT, IT, ET) of the specimens concerned is denoted and reference sequences for phylogenetic purposes (RS). Posterior probabilities ≥ 90 are displayed followed by bootstrap values greater than 60% for the maximum likelihood (ML) analyses conducted. Taxa in bold were sequenced for the current study.

**Figure 4.**
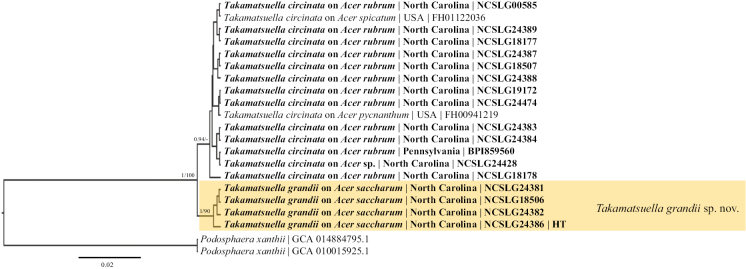
Bayesian maximum clade credibility tree of the concatenated ITS+28S+*GAPDH*+IGS+*TUB* regions of taxa in the genus *Takamatsuella*. Type status (HT, ET) of the specimens concerned is denoted. Posterior probabilities ≥ 90 are displayed followed by bootstrap values greater than 70% for the maximum likelihood (ML) analyses conducted. Taxa in bold were sequenced for the current study.

**Figure 5.**
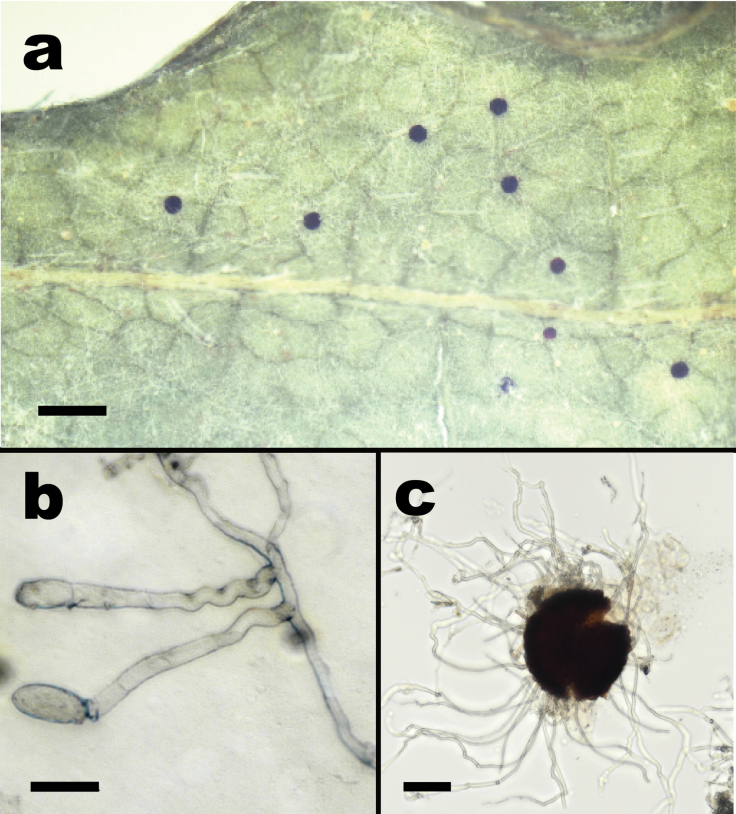
*Erysipheamphicarpaeicola* sp. nov. (**a, c** from NCSLG 22551 **b** from NCSLG25141) **a** habit, on leaves of *Amphicarpaeabracteata***b** conidiophores with conidia **c** split chasmothecium showing mycelioid appendages. Scale bars: 0.5 mm (**a**); 25 µm (**b**); 30 µm (**c**).

**Figure 6.**
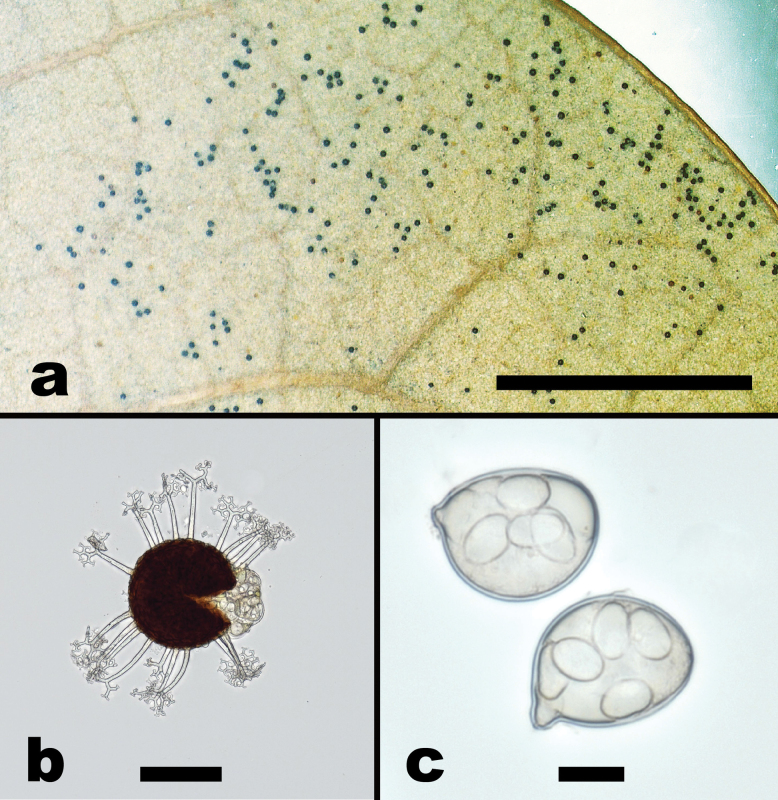
*Erysiphequercus-virginianae* sp. nov. (based on NCSLG 18481) **a** habit, on leaves of *Quercusvirginiana***b** split chasmothecium showing apically branched appendages and emerging asci **c** close-up of asci with ascospores. Scale bars: 4 mm (**a**); 100 µm (**b**); 20 µm (**c**).

**Figure 7.**
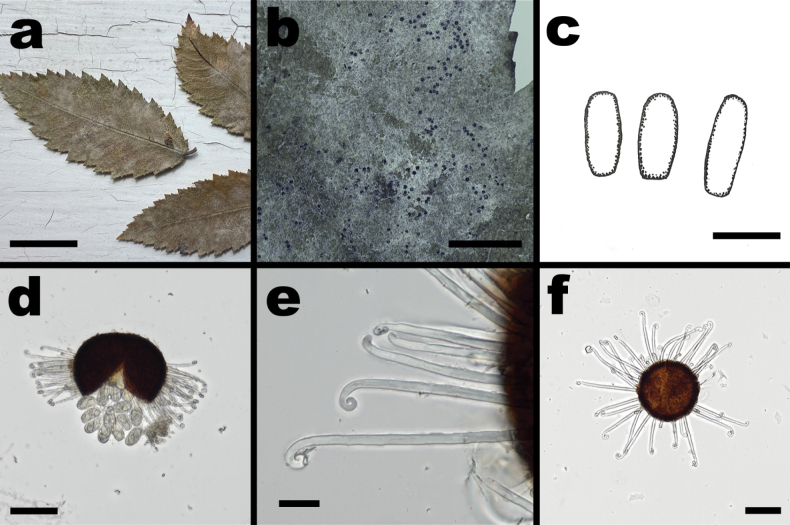
*Erysipheulmi-alatae* sp. nov. (**a, b, d, f** from NCSLG 18204 **c** from NCSLG 24391 **e** from NCSLG 24393) **a** mycelium on leaves of *Ulmusalata***b** habit, on leaves of *Ulmusalata*, showing clustered chasmothecia **c** conidia (drawing by S. LaGreca) **d** split chasmothecium showing numerous appendages with circinate ends, and asci with ascospores **e** appendages, immature (top) and mature (bottom) **f** whole chasmothecium. Scale bars: 2 cm (**a**); 4 mm (**b**); 30 µm (**c**); 20 µm (**b, e**); 75 µm (**f**).

DNA was successfully extracted, amplified, and sequenced from 219 of 299 (73% success rate) specimens (Suppl. material 1). All sequences were deposited in GenBank and all specimen data are now available on MycoPortal (Suppl. material 1). Based on sequence analyses, 60 species in 10 genera collected from 134 host plant species were represented in the sample. Ninety-five percent of the specimens were collected from North Carolina (Fig. 1). Phylogenetic analyses resulted in new assignments and determinations for 84% of the specimens sequenced and revealed four undescribed species (Figs 2–7). Most sequenced specimens were collected between 1965 and 2012. An additional observation is that we extracted DNA from and sequenced the ITS+28S region of a *Brasiliomyces* specimen several times due to uncertain results. The ITS region consistently displayed complex electropherograms with multiple bands in certain regions, likely resulting from intragenomic variation in this species (Bradshaw et al. 2023c).

### ﻿Taxonomy

#### ﻿ Erysiphe amphicarpaeicola

Taxon classificationAnimaliaHelotialesErysiphaceae

M. Bradshaw sp. nov.

122556AB-1127-5C5F-9A5A-7D663D1F982D

858351

Fig. 5

##### Etymology.

Epithet composed of the name of the host genus and the Latin-derived suffix “-cola” (dweller).

##### Diagnosis.

Morphologically distinguished from *Erysipheglycines* by having chasmothecia with shorter appendages, up to twice as long as the chasmothecial diameter (versus up to seven times as long as the diameter in *E.glycines*), and phylogenetically by forming a distant highly supported clade.

##### Type.

**USA** • North Carolina, Ashe County, near West Jefferson, on leaves of *Amphicarpaeabracteata*, along Bluff Mountain logging road, 36°23'26.9"N, 81°33'21.2"W, 14 September 2024, J. Thompson (NCSLG 25124—holotype). Ex-holotype sequence: PV416665 (ITS), PV472002 (*GAPDH*), PV471964 (*GS*).

##### Description.

***Mycelium*** amphigenous, forming effuse, arachnoid, whitish patches; ***hyphae*** branched, often at right angles, septate, hyaline, thin-walled, smooth, 3–5 µm wide; hyphal appressoria solitary, nipple-shaped to lobate, 4–10 µm wide; conidiophores 65–80 µm long, septate at base, foot cells 24–42 µm long and 6–8 µm wide, straight to usually somewhat flexuous, sinuous, followed by 1–2 shorter cells, about 15–20 × 7–8 µm ***conidia*** formed singly, cylindrical-doliiform, 23–35 × 10–12 µm, germination not seen. ***Chasmothecia*** scattered, subglobose to globose, dark brown, 90–154 × 104–157 µm in diameter; ***peridium*** cells irregularly polygonal, 6–13 × 10–19 µm; ***appendages*** in the lower half of the chasmothecium, number variable, few to numerous, mycelioid, often interwoven with the mycelium and with each other, sometimes poorly developed and hard to distinguish from the mycelial hyphae, length variable (up to two times the diameter of the chasmothecium), thin-walled, smooth, narrow (up to 4 µm wide), septate, hyaline; ***asci*** 4–6 per chasmothecium, 63–70 × 38–45 µm, oblong-ellipsoid, short-stalked, 6-spored; ***ascospores*** ellipsoid-ovoid, 20–22 × 11–12 µm, colorless.

##### Additional specimens examined.

(all on leaves of *Amphicarpaeabracteata*): **USA** • North Carolina, Macon County, Cashiers, along roadside, 35°00'59"N, 83°07'37"W, 2024, June 2024, J. Thompson 100 (NCSLG 25142); • Shortoff Mountain along trail, 35°00'59"N, 83°07'37"W, June 2024, J. Thompson 101. (NCSLG 25141); • Macon County, Highlands, on leaves of *Amphicarpaeabracteata* along roadside, 16 September 1975, L.F. Grand 2095 (NCSLG 22551).

##### Substrate/host.

*Amphicarpaeabracteata*.

##### Distribution.

North America (Canada, USA), probably widespread.

##### Notes.

Amano (1986) listed *Amphicarpaeabracteata* as host of *Erysiphecommunis* from Canada and the United States. Braun and Cook (2012) assigned North American *Erysiphe* specimens on *A.bracteata* to *E.glycines*, an Asian species that occurs on *Glycine* spp. and *Amphicarpaeaedgeworthii*. This decision was based on the morphological similarity between Asian and North American specimens. Sequences retrieved from Asian collections on *Glycine* spp. and *Amphicarpaeaedgeworthii* form a strongly supported species clade within the basal *Uncinula* lineage within *Erysiphe* (Bradshaw et al. 2023b). The North American *E.amphicarpaeicola* clade also clusters in the *Uncinula* lineage, far from *E.glycines* (Fig. 2). Based on the examined specimens, chasmothecia on *A.bracteata* differ from *E.glycines* chasmothecia in having shorter appendages, up to twice as long as the chasmothecial diameter (versus up to seven times as long as the diameter).

#### ﻿ Erysiphe quercus-virginianae

Taxon classificationAnimaliaHelotialesErysiphaceae

M. Bradshaw sp. nov.

4DCFE857-5543-5F61-8304-05D421F5FF72

858352

Fig. 6

##### Etymology.

Epithet derived from the name of the host plant, *Quercusvirginiana*.

##### Diagnosis.

Morphologically close to *Erysipheabbreviata* s. lat., but differing by forming much larger chasmothecia, 116–159 µm diam., with up to 20 appendages, and 4–8-spored asci. Phylogenetically well-distinguished from *E.abbreviata* and all other North American *Erysiphe* spp. on oaks by forming a highly supported clade.

##### Type.

**USA** • North Carolina, Wake County, J.C. Raulston Arboretum, NC State University, 4415 Beryl Road, Raleigh, on *Quercusvirginiana* planted in the arboretum, 35°47.687.10'N, 78°41.97100'W, 149 m alt., 9 November 2011, L.F. Grand s.n. (NCSLG 18481—holotype). Ex-holotype sequence: OR424987 (ITS+28S), OR427493 (*CAM*), OR427579 (*GAPDH*), OR427663 (*GS*), OR427727 (*RPB2*), OR427793 (*TUB*).

##### Description.

***Mycelium*** and ***anamorph*** not seen. ***Chasmothecia*** scattered to gregarious among trichomes on abaxial leaf surfaces, subglobose to globose, 116–159 × 123–144 µm; ***peridium*** cells conspicuous, brown, irregularly polygonal, 12–21 × 7–14 µm; ***appendages*** 8–20, equatorial, stiff, straight to somewhat curved, aseptate, hyaline, 60–125 µm long, relative length usually about 0.5–1 times the chasmothecial diameter or somewhat shorter, 4–7 µm wide [widest at base], ***apices*** 4–5 × regularly dichotomously branched not strictly in one dimension, tips of the ultimate branchlets recurved; ***asci*** 5–8 per chasmothecium, obovoid, saccate, short-stalked, 55–75 × 40–60 µm, walls up to 3 µm thick, 4–8-spored; ***ascospores*** ellipsoid-ovoid, hyaline, 15–25 × 8–13 µm.

##### Additional specimen examined.

**USA** • Florida, Broward County, Fort Lauderdale, on *Quercusvirginiana*, 2022, M.J. Bradshaw s.n. (NCSLG 24888).

##### Substrate/host.

*Quercusvirginiana* (Quercussubgen.Quercussect.Virentes; Manos and Hipp 2021).

##### Distribution.

North America (USA, Florida, North Carolina).

##### Notes.

The new species, *Erysiphequercus-virginianae*, is morphologically similar to the morphology-based circumscription of *E.abbreviata* in Braun and Cook (2012), especially with regard to the number and length of the chasmothecial appendages. However, *E.abbreviata* is characterized by having smaller chasmothecia, 70–110 µm diam, with fewer, 3–6-spored, asci (3–6), and somewhat larger ascospores, 20–32 × 13–21 µm when mature. Bradshaw et al. (2025d) published a phylogenetic-taxonomic revision of North American *Erysiphe* spp. on oaks, including a re-assessment of *E.abbreviata*. A high degree of co-evolution between *Erysiphe* and *Quercus* species was revealed in that study and led to the introduction of multiple new species as well as emended circumscriptions of several species. Based on phylogenetic examination, *E.abbreviata* is confined to hosts of Quercussubgen.Quercus sect. Quercussubsect.Prinoideae. Hence, it is plausible that *E.quercus-virginianae*, on a host of another section (*Virentes*) represented another, undescribed species. *Quercusvirginiana* and all other oak species assigned to sect. Virentes are to our knowledge not hosts of *E.abbreviata* (not listed as hosts in Braun and Cook 2012). Amano (1986) listed *Microsphaeraalni* and *M.extensa* on *Q.virginiana* from North America. *Erysipheextensa* is a morphologically distinct species with very long chasmothecial appendages (Braun and Cook 2012; Bradshaw et al. 2025d), whereas *E.quercus-virginianae* may be potentially hidden under reports of *M.alni* on *Q.virginiana*.

#### ﻿ Erysiphe ulmi-alatae

Taxon classificationAnimaliaHelotialesErysiphaceae

M. Bradshaw sp. nov.

55F4B9AF-EC92-59FB-8CD6-D5C14EEF9473

858353

Fig. 7

##### Etymology.

Epithet referring to the name of the type host, *Ulmusalata*.

##### Diagnosis.

*Erysipheulmi-alatae* is morphologically barely distinguishable from *E.macrospora*, but can be distinguished by the host and the fact that it phylogenetically forms a separate, highly supported clade.

##### Type.

**USA** • North Carolina: Wake County, Beaverdam Campground, Falls Lake, in upland hardwood-pine forest, on leaves of *Ulmusalata*, 3 November 2008, *L.F. Grand* 2095 and *C.A. Vernia* (NCSLG 18204 – holotype). Ex-holotype sequence: PV416510 (ITS), PV409586 (IGS).

##### Description.

***Mycelium*** in persistent, creamy-white patches, almost entirely on adaxial leaf surfaces; ***hyphae*** branched, often at right angles, septate, hyaline, 3–5 µm wide; hyphal appressoria solitary or in opposite pairs, lobed; ***conidia*** formed singly, cylindrical-doliiform, 34–40 × 11–18 µm. ***Chasmothecia*** scattered to gregarious, dark brown, subglobose to globose, 130–178 µm in diameter; ***peridium cells*** irregularly polygonal, 7–13 × 10–21 µm; ***appendages*** numerous, number variable (40+), hyaline, aseptate, ± equatorial, 80–145 × 5–9 µm, mostly shorter than the chasmothecial diameter, width ± equal throughout, walls smooth, uniformly thickened from base to tip, ***apices*** uncinate to circinate when mature, uncinate-circinate apex not enlarged, circinate apices 10–15 µm across (appendages shorter, stiffer, and with pointed ends when immature); ***asci*** 10–25 per chasmothecium, obovoid, saccate, short-stalked, 48–70 × 23–24 µm, walls up to 3 µm thick, 2-spored; ***ascospores*** ellipsoid-ovoid to slightly teardrop-shaped, 22–30 × 11–17 µm, hyaline.

##### Additional specimens examined.

(all on leaves of *Ulmusalata*): USA • North Carolina, Cabarrus County, Concord, 15 October 1972, R.L. Forster 63 (NCSLG 24392); • Wake County, Cary, 7 July 2008, Y. Weimin s.n. (NCSLG 17649); • NC State University campus, Raleigh, 27 September 1972, A.J. Julius s.n. (NCSLG 24393); • Miner Presbyterian Church, New Bern Avenue, Raleigh, 24 September 1978, R. Sohn s.n. and G. Emberger (NCSLG 24391).

##### Substrate/host.

*Ulmusalata*.

##### Distribution.

(based on specimens deposited in North American herbaria as ‘*Erysiphemacrospora*’ or ‘*Uncinulamacrospora*’ on *Ulmusalata*): North America (USA: Alabama, Florida, Georgia, Illinois, Indiana, Mississippi, North Carolina, Oklahoma, South Carolina, Tennessee, Texas).

##### Notes.

*Erysiphe* (*Uncinula* lineage) on *Ulmusalata* was previously assigned to *Uncinulamacrospora* and *Erysiphemacrospora*, respectively (Salmon 1900; Braun 1987; Braun and Cook 2012). However, in the first phylogenetic examinations of *E.macrospora*, Bradshaw et al. (2023b) revealed the paraphyly of this species. Sequences obtained from the type host, *Ulmusamericana*, as well as on *U.rubra* (= *U.fulva*) and *U.pumila*, formed a well-supported clade, with a sequence retrieved from *U.alata* clustering outside in sister position. Now, additional sequences are available and confirm the *Erysiphe* on *U.alata* as a distinct, cryptic, sister species. The genetic similarity between *E.macrospora* and *E.ulmi-alatae* in multiple loci is relatively low (~95%), which supports the description of a separate species. The two species are morphologically barely distinguishable, i.e., they can only be differentiated by their sequence differences and different hosts. The separation of *E.macrospora* s. lat. into two species, based on its host species, is not surprising. According to the current phylogenetic-taxonomic division of the genus *Ulmus* (Whittemore et al. 2021), the type species, *Ulmusamericana*, pertains to Ulmussubgen.Oreopteleaesect.Blepharocarpus, whereas *U.alata* is assigned to Ulmussubgen.Oreopteleaesect.Chaetoptelea. *Ulmuscrassifolia* and *U.thomasii* (= *U.racemosa*) are two additional elm species known to be hosts of *E.macrospora* s. lat. (Braun and Cook 2012) that belong to sect. Chaetoptelea. It can be assumed that these elm species also pertain to the host range of *E.ulmi-alatae*, which is, yet, unproven by means of sequence analyses. Additionally, *Ulmusrubra* (= *U.fulva*), a species pertaining to Ulmussubgen.Ulmussect.Ulmus, is a proven host of *E.macrospora*, suggesting a wider host range of this species. The occurrence of *E.ulmi-alatae* on *U.alata* likely follows the distribution of its host species in the Southeastern and Central USA.

#### ﻿ Phyllactinia liriodendri

Taxon classificationAnimaliaHelotialesErysiphaceae

U. Braun, in Braun and Cook, Taxonomic Manual of the Erysiphales (Powdery Mildews): 260. 2012.

4858E784-03C5-5049-92C9-B716E8697E3B

##### Type.

**USA** • Pennsylvania, Centre County, State College Campus, on *Liriodendrontulipifera*, 1889, W.A. Buckhout (BPI 859705—holotype).

##### Epitype.

(designated here, MycoBank, MBT10025444): **USA** • North Carolina, Wake County, Raleigh, on *Liriodendrontulipifera*, November 1972, L. Lazo s.n. (NCSLG22914). Ex-epitype sequences: PQ585171 (ITS), PQ589086 (*TUB*).

##### Notes.

*Phyllactinialiriodendri* was included in phylogenetic-taxonomic studies recently published by Bradshaw et al. (2025b). The authors suggested that sequences from specimens of this species form highly supported species clades in concatenated, ITS+28S, and *TUB* analyses, confirming the status of *P.liriodendri* as a species within the morphologically poorly differentiated genus *Phyllactinia*, which requires sequence data for unequivocal identifications. *Ex typus* sequences are the best option to obtain and analyze reference sequences for phylogenetic-taxonomic purposes. However, the type of *P.liriodendri* from 1889 is very old and sequencing was not attempted. In such cases, epitypification with ex-epitype sequences is the method of choice.

#### ﻿ Phyllactinia

Taxon classificationAnimaliaHelotialesErysiphaceae

sp. on Carpinus caroliniana

8E4A0E3D-3429-504C-B82B-6817727CB8F3

Fig. 8

##### Description.

***Mycelium*** amphigenous, mostly effuse but sometimes forming thin white patches; ***hyphae*** branched, septate, hyaline, thin-walled, smooth; ***hyphal appressoria*** not observed; anamorph not observed. ***Chasmothecia*** scattered, sphaeroid or nearly so, 180–200 µm in diameter; ***peridium*** cells large, light brown, irregularly polygonal, up to 30 µm long; ***appendages*** equatorial, between 4 and 10 per chasmothecium, aseptate, hyaline, up to 1.5 times the chasmothecial diameter, 5–7 µm wide, straight or almost so, rigid, acicular with bulbous swelling at the base (up to 40 µm wide); ***asci*** absent or poorly formed, indistinct, yellowish; ***ascospores*** not observed.

**Figure 8.**
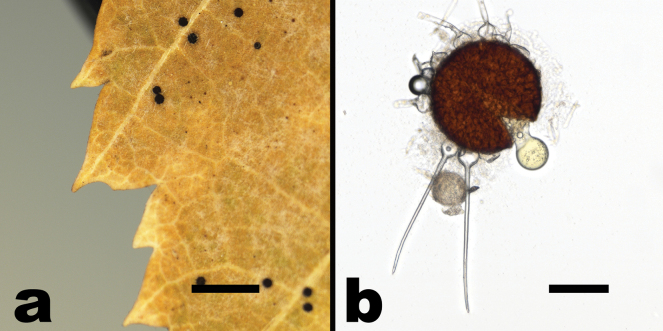
*Phyllactinia* sp. (based on NCSLG 17103) **a** habit, on leaves of *Carpinuscaroliniana***b** split chasmothecium showing peridium cells, emerging immature asci, and appendages with bulbous bases. Scale bars: 2 mm (**a**); 100 µm (**b**).

##### Specimen examined.

**USA** • North Carolina, Carteret County, Theodore Roosevelt State Natural Area, Bogue Banks, town of Pine Knoll Shores, on leaves of *Carpinuscaroliniana*, 26 November 2004, L.F. Grand and C.A. Vernia (NCSLG 17103).

##### Notes.

Owing to the morphological similarity, Braun and Cook (2012) assigned *Phyllactinia* on *Carpinuscaroliniana* in North America tentatively to *P.carpini*. The Asian *P.carpinicola* is morphologically readily distinguishable from *P.carpini* by having apiculate conidia (Braun and Cook 2012), and it is phylogenetically distinct (Takamatsu et al. 2008; Bradshaw et al. 2025b). Sequences obtained from the *Phyllactinia* specimen collected in North Carolina on *C.carolinana* clearly showed that this powdery mildew fungus does not pertain to the European *P.carpini*. However, it is premature to introduce a new species for this *Phyllactinia*. There are only sequences obtained from a single immature specimen, without the anamorph and immature chasmothecia without mature asci and ascospores. Additional sequenced collections are needed. A further problem refers to *P.carpini*, from which there is only one sequence retrieved from *Carpinusorientalis* available (Bradshaw et al. 2025b) which is not the type host of this species. The North American *Phyllactinia* on *Carpinuscaroliniana* is probably an undescribed species, but additional sequence data from mature specimens are needed, as well as an examination of European specimens on *Carpinusbetulus*, the type host of *P.carpini*.

#### ﻿ Takamatsuella grandii

Taxon classificationAnimaliaHelotialesErysiphaceae

M. Bradshaw sp. nov.

393024AF-043A-503C-A5A8-AAE92FB9805E

858354

Fig. 9

##### Etymology.

Epithet in honor of NCSU mycologist Larry F. Grand.

**Figure 9.**
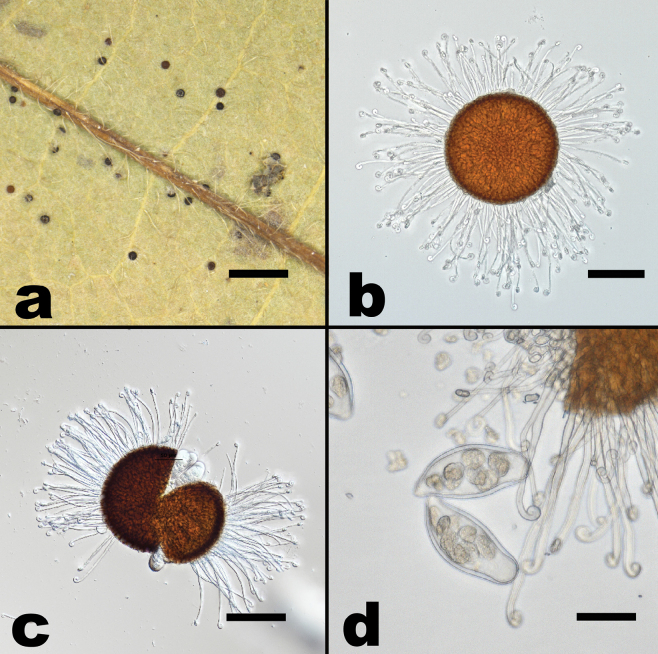
*Takamatsuellagrandii* sp. nov. (**a** From NCSLG 18506 **b–d** from NCSLG 24386) **a** habit, on leaves of *Acersaccharum***b** chasmothecium showing peridium cells and appendages with circinate ends **c** split chasmothecium showing appendages and emerging asci **d** close-up of asci with ascospores. Scale bars: 1 mm (**a**); 100 µm (**a, c**); 10 µm (**d**).

##### Diagnosis.

*Takamatsuellagrandii* differs morphologically from *T.circinata* in having appendages with walls uniformly 3 µm thick, and genetically by forming a highly supported clade.

##### Type.

**USA** • North Carolina, Wake County, Ligon Street, Raleigh, on leaves of *Acersaccharum*, 5 November 1980, M. Daykin s.n. (NCSLG 24386—holotype). Ex-holotype sequences: PV416651 (ITS), PV409641 (IGS).

##### Description.

***Mycelium*** on abaxial surfaces of leaves, effuse, thin, arachnoid, grayish white; ***hyphae*** dichotomously branched, hyaline, thin-walled, smooth, septate; ***hyphal appressoria*** nipple-shaped; ***anamorph*** not seen. ***Chasmothecia*** scattered to +/- gregarious, depressed globose, 120–170 µm in diameter; ***peridium cells*** irregularly polygonal, light brown, 7–15 × 14–19 µm; ***appendages*** very numerous, up to 150 per chasmothecium, arising below the equator, stiff to flexuous, simple, apices tightly uncinate to circinate, not enlarged, about 0.3–1 times as long as the chasmothecial diameter, uniformly 2–6 µm wide and walls uniformly 3 µm thick, hyaline, aseptate, smooth, thin-walled; ***asci*** up to 8 or more, clavate-saccate, 70–90 × 25–40 µm, usually stalked, wall uniformly 3 µm thick, 8-spored; ***ascospores*** ellipsoid-obovoid, 10–25 × 9–20 µm, hyaline.

##### Additional specimens examined.

**USA** • North Carolina, Wake County, Schenck Research Forest, Raleigh, on leaves of *Acersaccharum*, in floodplain along stream, 35°48.958'N, 78°44.020'W, 165 m alt., 26 October 2011, L.F. Grand s.n. (NCSLG 18506); • Wake County, Schenck Research Forest, Raleigh, on leaves of *Acersaccharum* (= *A.barbatum*, ≡ A.saccharumvar.barbatum, ≡ A.saccharumf.barbatum; = *A.dasycarpum*), 6 October 1998, G. Blosser 32 (NCSLG 24381).

##### Substrate/host.

*Acersaccharum*.

##### Distribution

(based on specimens on *Acersaccharum* deposited in North American herbaria): North America (USA: Indiana, New York, New Hampshire, North Carolina, Pennsylvania).

##### Notes.

*Takamatsuellagrandii* is an undescribed cryptic species infecting different *Acer* species to those of *T.circinatum*. The host of the type specimen of *T.circinata* is Acer*spicatum* (Acersect.Spicata) (Braun and Cook 2012). Additional *Acer* spp. cited as host species of *T.circinata* are *A.glabrum* (unresolved name), *A.nigrum* (Acersect.Acerser.Saccharodendron), *A.pensylvanicum* (Acersect.Macranthum), *A.rubrum* (Acersect.Rubra), *A.saccharum* (Acersect.Acerser.Saccharodendron), and *A.saccharinum* (Acersect.Rubra); subgeneric affiliations according to Davis (2021). Phylogenetic analyses of specimens of *Takamatsuella* on *Acersaccharum* revealed the existence of a cryptic species on this host, now referred to as *T.grandii*. The new species presented here is morphologically very close to *T.circinata* but differs in having appendages with walls uniformly 3 µm thick (versus thin-walled or only thickened at the base). The affinity of *Takamatsuella* species on the other host species listed above remains unclear since they belong to different sections of *Acer*. Based on the currently available sequences, as well as the high degree of co-evolution within this group of powdery mildews, it can be assumed that the *Takamatsuella* species on *Acernigrum* might be *T.grandii*, whereas specimens on *A.rubrum* and *A.saccharinum* (sect. Rubra) are expected to be part of the host range of *T.circinata*, pending molecular confirmation.

## ﻿Discussion

Phylogenetic analyses of DNA from 220 herbarium specimens collected predominantly in central North Carolina from the 1970s through the early 2000s yielded four undescribed powdery mildew fungi, as well as multiple, additional, potentially undescribed species that require further, comprehensive examination. Three of the new species are in the genus *Erysiphe* (the most speciose genus in the *Erysiphaceae*), and one in *Takamatsuella*. We also detected a *Phyllactinia* sp. on *Carpinuscaroliniana* and multiple *Erysiphe* spp. infecting *Quercus* spp. that probably represent additional, undescribed species requiring further investigation. In total, approximately 84% of the powdery mildew holdings in NCSLG had to be re-determined at the species or genus rank based on our molecular phylogenetic analyses (Suppl. material 1).

All taxonomic novelties revealed by the molecular phylogenetic examinations presented here are cryptic species within known, long-recognized powdery mildew species. Specifically, for *Erysipheamphicarpaeicola*, the host of the type specimen (*Amphicarpaeabracteata*) was thought to be within the host range of the Asian *E.glycines*; for *E.ulmi-alatae*, the host of the type specimen (*Ulmusalata*) was thought to be within the host range of *E.macrospora*; and with regards to *Takamatsuellagrandii*, the host of the type specimen (*Acersaccharum*) was assumed to be within the host range of *T.circinata* s. lat. (Braun and Cook 2012). Additionally, *E.quercus-virginianae* on *Quercusvirginiana* agrees with the morphological species concept of *E.abbreviata* in Braun and Cook (2012). These results underscore the importance of revising previous morphological species concepts, primarily when broader host ranges and/or wider distributions, beyond continents, are involved. Furthermore, using ex type sequences of taxa as reference sequences for phylogenetic-taxonomic purposes is crucial. The application of sequences obtained from non-type specimens not confirmed by ex type sequences, may lead to erroneous conclusions in complexes of morphologically similar species or taxa with a high degree of cryptic speciation. However, type collections are sometimes too old to generate high-quality sequences for analyses, as exemplified by *Phyllactinialiriodendri*. Designation of epitypes derived from recently collected material can help to mitigate such problems. In such cases, specimens deposited in herbaria can be valuable resources for helping to choose appropriate epitypes.

## ﻿Conclusion

The trend of discarding or providing reduced support for herbarium collections has become increasingly prevalent. This is driven by a combination of financial constraints, space limitations, and shifting priorities within academic institutions (Davis 2024). Digitization is helpful but is not a solution for many issues—cryptic species, for example, cannot be detected through the use of an image. Despite their historical importance for conservation, education, and taxonomic research, many herbaria are facing obsolescence as both physical and digital management become more burdensome. The rise of digital herbarium initiatives and pressure to repurpose or downsize institutional spaces have led to the deaccessioning of valuable collections, posing significant risks to the continuity of plant biodiversity research and the preservation of historical botanical records (Thiers et al. 2024). The discovery of four undescribed powdery mildew species in the present study underscores the urgent need to safeguard these irreplaceable scientific resources by increasing both their accessibility and use. As exemplified in the present study, the possibility to sequence herbarium specimens highlights the enormous potential of preserved material for documentation of biodiversity. Previous identifications can be verified and corrected if found to be inaccurate, which is essential in the light of the high degree of outdated and erroneous identifications in herbarium specimens, particularly for microscopic fungi. Furthermore, our results underline the importance of depositing herbarium specimens, and linking their sequence and annotated specimen data to repositories (i.e. GenBank and MycoPortal) (Conrad et al. 2014) and data networks (iDigBiio and GBIF). Depositing voucher and reference specimens in officially recognized herbaria is a critical element for satisfying the “reproducibility” criterion, one of the key tenets of the scientific method, for results reported in peer-reviewed scientific journals.

## Supplementary Material

XML Treatment for Erysiphe amphicarpaeicola

XML Treatment for Erysiphe quercus-virginianae

XML Treatment for Erysiphe ulmi-alatae

XML Treatment for Phyllactinia liriodendri

XML Treatment for Phyllactinia

XML Treatment for Takamatsuella grandii

## ﻿Additional information

### Adherence to national and international regulations

All the fungal strains used in this study have been legally obtained, respecting the Convention on Biological Diversity (Rio Convention).

### Author ORCIDs

Andrew Paul https://orcid.org/0009-0000-3499-4080

Michael Bradshaw https://orcid.org/0000-0002-7133-4679

### Data availability

All of the data that support the findings of this study are available in the main text or Supplementary Information.

## Supplementary materials

Supplementary material 1 List of taxa, hosts, vouchers, collection localities, and GenBank accession numbers of the specimens examined in the current study Data type xlsx https://binary.pensoft.net/file/1349529This dataset is made available under the Open Database License (http://opendatacommons.org/licenses/odbl/1.0/). The Open Database License (ODbL) is a license agreement intended to allow users to freely share, modify, and use this Dataset while maintaining this same freedom for others, provided that the original source and author(s) are credited.Scott LaGreca, Uma Crouch, Andrew Paul, Jacklyn Thomas, Jake Thompson, Christian Shaw, Marc A. Cubeta, Uwe Braun, Michael Bradshaw
